# When New Experience Leads to New Knowledge: A Computational Framework for Formalizing Epistemically Transformative Experiences

**DOI:** 10.1162/opmi_a_00168

**Published:** 2024-11-01

**Authors:** Joan Danielle K. Ongchoco, Isaac M. Davis, Julian Jara-Ettinger, L. A. Paul

**Affiliations:** Department of Psychology, The University of British Columbia; Department of Psychology, Yale University; WuTsai Institute, Yale University; Department of Philosophy, Yale University; Cognitive Science, Yale University; Munich Center for Mathematical Philosophy, Ludwig Maximilian University of Munich, Munich, Germany

**Keywords:** computational framework, epistemic change, learning, transformative experience

## Abstract

The discovery of a new kind of experience can teach an agent what that kind of experience is like. Such a discovery can be epistemically transformative, teaching an agent something they could not have learned without having that kind of experience. However, learning something new does not always require new experience. In some cases, an agent can merely expand their existing knowledge using, e.g., inference or imagination that draws on prior knowledge. We present a computational framework, grounded in the language of partially observable Markov Decision Processes (POMDPs), to formalize this distinction. We propose that epistemically transformative experiences leave a measurable “signature” distinguishing them from experiences that are not epistemically transformative. For epistemically transformative experiences, learning in a new environment may be comparable to “learning from scratch” (since prior knowledge has become obsolete). In contrast, for experiences that are not transformative, learning in a new environment can be facilitated by prior knowledge of that same kind (since new knowledge can be built upon the old). We demonstrate this in a synthetic experiment inspired by Edwin Abbott’s *Flatland*, where an agent learns to navigate a 2D world and is subsequently transferred either to a 3D world (epistemically transformative change) or to an expanded 2D world (epistemically non-transformative change). Beyond the contribution to understanding epistemic change, our work shows how tools in computational cognitive science can formalize and evaluate philosophical intuitions in new ways.

## INTRODUCTION

Consider a colorblind person who, after an eye operation, sees red for the first time. They discover something new: they now know what it’s like to see red. Philosophers (Jackson, 1986; Lewis, 1990; Paul, 2014) have argued that the discovery of this type of “what it’s like” knowledge requires experience, for a person cannot know what it is like to see red without having the experience of seeing red. The change in experience brings a change in epistemic status. Our colorblind individual’s discovery of red experience gives them knowledge of what it is like to see red, manifested in part by a change in their abilities to perform certain tasks, such as the ability to imagine, distinguish between, and classify red things.^1^

Gaining new “what it’s like” knowledge does not always require new experience (for philosophical discussion, see Kind, 2020). For example, to know what it’s like to taste a novel combination of two familiar tastes may not require new experience, as the agent could infer this based on their prior experience. Consider the possibility of tasting an avocado-raspberry milkshake. Previous work suggests that retrieving memories of each of these can facilitate imaginative evaluation of the novel combination (e.g., Barron et al., 2013). If so, a person can projectively imagine or infer what the novel combination tastes like without having actually experienced it. In this sense, pre-existing knowledge of what it’s like to taste avocado, to taste raspberry, and to taste a milkshake can help us imaginatively infer the taste of an avocado-raspberry milkshake; intuitively, the agent projectively combines knowledge they already have. In contrast, for a formerly colorblind individual to discover what it is like to see red, they must have direct experience of seeing red; the knowledge cannot be generated merely by combining elements of what is already known. In the “seeing red” case, the relevant new knowledge is not accessible to the individual (neither by imagination nor by inference).

We hypothesize that the difference here stems from differences between the kinds of human experiences involved. Taking a page from the metaphysics of natural kinds, we can think of experiences in terms of natural experience kinds, grouping experiences into kinds based on their (natural) experiential similarities. One can have the new experience of seeing red, and this is of a different kind from previous experiences of seeing green. Conversely, one can have the new experience of tasting an avocado-raspberry-milkshake, but this is not relevantly different from previous experiences of tasting avocados, tasting raspberries, and tasting milkshakes. The latter is not a new kind of experience; it is merely a new variant of a familiar species of experience.

For this reason, experience seems to be necessary for our colorblind individual to know what it is like to see red, while experience does not seem to be necessary (for a person with prior experience of tasting avocados, raspberries, and milkshakes) to know what it’s like to taste an avocado-raspberry-milkshake. If so, experience can be necessary, in certain contexts, for an individual to learn new things.

We can describe the discovery of a new kind of experience and the distinctive type of learning it brings as “epistemically transformative” learning (Paul, 2014). In this paper, we propose a computational framework, grounded in the language of Partially Observable Markov Decision Processes (POMDPs), for identifying when the discovery of a new kind of experience triggers epistemically transformative learning. In particular, we propose that epistemically transformative learning (where the relevant knowledge could not have been extrapolated from prior knowledge, so requires a new kind of experience) leaves a distinct and measurable “signature” that distinguishes it from non-transformative learning (where the relevant knowledge could have been extrapolated, at least partially, from prior knowledge). Here we present an initial validation of this theory with a pair of case studies. Importantly, we do *not* aim to provide an exhaustive definition of epistemic transformation, nor do we provide necessary or sufficient conditions for learning to be considered epistemically transformative. Rather, we argue that certain learning patterns are frequently indicative of epistemically transformative learning, and demonstrate how these patterns can be formalized and quantified in a computational framework.

The remainder of this paper is organized as follows: first, we provide a brief overview of POMDPs and motivate how they can capture the relevant markers of epistemic transformation. We present our formal framework and describe how it can distinguish epistemically transformative from non-transformative learning. We then apply the framework to a pair of case studies inspired by Edwin Abbott’s *Flatland* (Abbott, 1987), which follows an agent living in a two-dimensional world who suddenly acquires the ability to perceive in 3D (Modeling New Experiences in POMDPs section). Through a series of simulations, we demonstrate how this epistemically transformative learning has a particular “signature” that distinguishes it from non-transformative learning (Evidence for Restructuring? section). We then propose a method for analyzing how exactly the agent’s knowledge is changed (What Gets Restructured? section), and what distinguishes this change in knowledge between the transformative and non-transformative cases. We then assess this computational framework against human intuitions (Validating the Computational Results With Human Intuitions section).

### POMDPs as a Framework for Epistemic Change

To formalize the distinction between epistemically transformative and non-transformative learning, we used partially observable Markov decision processes (or POMDPs), a framework for modeling how agents act in the world under uncertainty (Cassandra, 1998; Sutton & Barto, 1998). We begin with a brief review of POMDPs (a more extended introduction can be found in Cassandra, 1999) and then turn to the way we will use POMDPs in the current study to explore how and when new experiences lead to changes in knowledge.

#### Brief Introduction to POMDPs.

At a high level, POMDPs represent interactive environments where an agent takes extended sequences of actions that typically incur costs (e.g., when moving in space), generate rewards (e.g., when completing a goal), and affect the agent’s beliefs about the state of the world (by providing new information based on, e.g., what the agent can and cannot see). The space of possible world states is represented as a discrete finite state space *S*, where each state represents a full configuration of the world (such that the environment is always in one of the states from the state space). The agent’s possible behaviors are represented as a discrete finite action space *A*, and the dynamics of how actions affect the world are represented by a transition function *T* that specifies how the world probabilistically changes from one state to another given a particular action (e.g., taking action ‘eat an apple’ in a world state where the agent is standing next to the apple is likely to transition the environment to the world state where the apple is now gone).

Critically, a POMDP agent has only partial access to the true state of the world (e.g., an agent might know that there are two boxes in the room, but not know what is inside each box). We can represent an agent’s beliefs about the state of the world as a probability distribution over the state space *S*, such that a uniform distribution over *S* captures full ignorance while concentrating all the probability on a single state captures full confidence. This representation is dynamic and changes as the agent interacts with the world. To achieve this, POMDPs formalize a discrete finite observation space *O*, which specifies all the possible pieces of information that an agent might receive, and an observation function that defines what information an agent receives as a function of world states and actions (e.g., taking action ‘open box’ could produce an ‘empty’ or ‘full’ observation, depending on whether there is an object in the box or not). As the agent receives information about the world, it updates its beliefs rationally using Bayesian inference, by considering what states are most likely to explain the observations.

Finally, the agent’s actions in the world produce costs and rewards. For instance, an agent could incur a cost for every movement it takes and obtain a reward for every object it collects. Given these specifications (state space, action space, observation space, reward function, transition function, and observation function), it is possible to compute how an agent ought to behave to maximize its long-term rewards (with the addition of a future discount parameter that specifies how much to prioritize short-term rewards over long-term ones). This normative plan, called a *policy*, captures the agent’s knowledge of how to navigate the world to achieve its goals.

Because the term ‘knowledge’ may mean different things across disciplines, we note that the POMDP framework involves two distinct kinds of epistemic representations: the first is the agent’s representation of the possible states of the world (i.e., how the world might be), encoded by the aforementioned probability distribution P(S) over world-states. The second is the agent’s representation of how best to navigate that world in pursuit of a goal, encoded by the aforementioned policy. The “knowledge change” we aim to capture in our framework refers to the latter: given a change in the way the agent experiences the world, the key question is how the agent adapts their strategy for navigating the world (i.e., their policy). To avoid confusion, and to maintain consistency with standard POMDP terminology, we refer to the agent’s representation of possible world-states (P(S)) as *beliefs*, and the representation of how to navigate the world (policy) as *knowledge*.

#### Capturing Epistemic Change in POMDPs.

Returning to our question of interest, our goal is to specify how a new kind of experience brings a change in knowledge. To do this, we propose using POMDPs to measure the degree to which an agent’s prior knowledge (i.e., the initial policy for solving the POMDP task) is useful for adapting to a new kind of experience.

In the context of POMDPs, we formalize a change in the agent’s experience (e.g., seeing a new color) as a change in the observation space (the experiences an agent can have). For example, for an agent who can only see in greyscale, a newfound ability to see color might correspond to an additional dimension of information in the agent’s world-state representation. This change in experience might enable new strategies previously unavailable to the agent (such as comparing two objects by color), leading the agent to adapt their policy to incorporate these new capacities. We equate this change in policy with a change in the agent’s knowledge. If this change in knowledge is *epistemically transformative*, then the agent’s initial policy (prior knowledge) should not be useful for learning the new policy—and thus, the new policy replaces the old policy. On the other hand, if a change in knowledge is *non-transformative*, the agent should be able to extrapolate the new policy (at least partially) from the old policy. Ultimately, this is a graded rather than binary distinction: a new experience may require either a complete overhaul of the agent’s knowledge or a slight modification of existing knowledge, but it may also involve just the replacement of some knowledge and minor adjustments to other knowledge.

To this end, we propose that whether a new experience is epistemically transformative is reflected by how much learning must occur to adjust the policy, in addition to which parts of the policy are being adjusted, after a new experience. Crucially, our proposal is *not* a raw measure of the total learning time required, but a *comparison* between the time required to learn the new policy using the original policy, relative to the time required to learn the new policy without the knowledge encoded in the original policy. That is, we will calculate the amount of learning needed to update an agent’s policy after a change in their experience, and we will compare it to the amount of learning that would be needed to build this new policy from scratch. Under this formulation, we can distinguish changes in experience that allow the agent to leverage their original policy when learning the new policy, with much of previous knowledge still applying, from those that require an effective replacement of the policy, such that the past knowledge carries no advantage relative to an agent learning how to act from scratch. We define a change in knowledge as “epistemically transformative” if the amount of learning required to update the policy is comparable to the amount of learning required to learn the policy from scratch. This is the empirical signature of epistemically transformative learning that we propose in our framework.

For example, consider again seeing red for the first time. We could first train an agent to fulfill its goals in a world where it cannot see red (i.e., train an agent to build a policy in a world without red experiences). Once the policy is built, we can give the agent access to red experience, and measure how much learning occurs when the agent updates its policy in light of this new kind of experience. To test whether this learning is epistemically transformative in the relevant sense, we compare the amount of learning that occurs in this update to the amount of learning required for an agent to build its initial policy in a world with the color red (using a “naive” agent, that is, one that has not been pre-trained in a world where it cannot see red). If the amount of learning in the case where the agent updates is comparable to the amount of learning in the case where the agent is naïve, this suggests that the knowledge change is epistemically transformative. Note that within this formulation, it is possible for an experience to be epistemically transformative even if the total learning time to acquire the new policy is not very large, and, conversely, it is possible for an experience to be non-transformative even if the adaption requires a significant amount of learning time. Thus, the key measure is not the total time required to learn the new policy, but the advantage to learning granted by the agent’s prior knowledge. We recognize that this does not eliminate all questions about how to identify epistemic transformation, but we take our framework to provide an important start towards grounding these questions in precise computational terms as opposed to giving the final word on the subject.

#### Measuring Learning Across Changes.

Building a POMDP policy is computationally expensive, and research in the last two decades has produced several different learning algorithms that learn approximate solutions efficiently (Hsu et al., 2008; Kurniawati et al., 2008; Ng & Jordan, 2013). Here we use the SARSOP algorithm (Successive Approximations of the Reachable Space under Optimal Policies; Kurniawati et al., 2008). SARSOP, like all POMDP solvers, consists of an iterative process that sequentially refines the policy until convergence (i.e., sequentially modifying the policy across iterations until the changes are small enough that they become inconsequential to the agent’s behavior). For our work, the critical element of SARSOP is that it only learns the subset of the policy needed to act under plausible knowledge states that a rational agent might have (whereas a complete POMDP policy specifies how an agent should act under any arbitrary knowledge state, including ones that an agent might never be under in practice). Using the SARSOP algorithm, we will use total computation time in training as a proxy for the amount of learning an agent has to go through: suppose that an agent has already learned a policy for solving a task in some POMDP environment. After learning this policy, the agent undergoes a change in how they experience that environment (e.g., gaining the ability to perceive a new color), and must adjust their policy to incorporate this new experience. The key question underlying our framework is, to what degree is the agent able to leverage their pre-existing knowledge (i.e., original policy) in order to adapt to this new experience (i.e., learn a new policy)? If the agent can simply make adjustments to their original policy in order to adapt (i.e., it does not require a lot of additional learning given the original policy), then the experience is considered non-transformative. If the original policy is useless, (i.e., the prior knowledge does not improve the agent’s learning time at all), then the experience is considered transformative.

In order to formalize this notion in the POMDP framework, we must translate this comparison into something that can be captured via the agent’s learning time. To this end, we compare the time it takes an agent to adapt their existing knowledge (original policy) to this new experience against the time it takes for the agent to learn a new policy without any useful prior knowledge. However, in this case, the latter agent learning to act in a new environment is *building* knowledge (i.e., constructing a policy from a new set of experiences, when there was no previous experience or knowledge), whereas the former agent obtaining a new experience after having learned to act in the environment is *modifying* existing knowledge (i.e., revising an existing policy). These two computations are not directly comparable, since the amount of knowledge that the agents are working with differs from the start. To address this, we always compared our main agent (i.e., the agent that learns to act in an environment and then undergoes an experience change) to a ‘scrambled knowledge agent’. This scrambled knowledge agent is identical to the main agent in all respects, such that we give the scrambled knowledge agent the same policy given to the main agent—except that we scrambled the information in the policy (i.e., randomly permuted the agent’s mapping from belief states to actions). This is designed to control for the concern about comparability: the “scrambled” agent is now initialized with the same amount of knowledge as the main agent trying to adapt their existing policy, but the content of this knowledge is useless (to mimic what it’s like to learn a policy without useful prior knowledge).

### Evaluating Our Computational Proposal

Our starting point for creating a world in which we could simulate transformative versus non-transformative experiences is Edwin Abbott’s *Flatland* (Abbott, 1987). Flatland follows an agent that lives in a two-dimensional world, such that any object in the world is perceived simply as a line. In this world, it is possible to identify objects by moving around them. For instance, a circle will be seen as a line of constant size as the agent circles it, whereas a square will be seen as a line that expands and contracts. Identifying objects in a 2D world therefore requires a type of behavioral competence that is not useful for agents living in a 3D environment. A 3D experience brings a new kind of visual experience and with it, new behavioral competencies. We characterize the move from a 2D to a 3D world as an epistemically transformative experience. We hypothesize that when the agent transitions into a 3D world, this new kind of visual experience leads to new knowledge, such that the agent’s prior knowledge (a policy for navigating the 2D environment) is minimally useful in learning to navigate the 3D environment. Our case study starts with an agent who learns to identify objects in a 2D environment, and considers what knowledge is added when a 3rd dimension is added (a transformative change), versus when new regions of the 2D environment are unlocked (a non-transformative change). We are comparing an agent who discovers a new kind of experience and thus a new kind of knowledge (3D knowledge) to an agent that is merely expanding or developing a familiar kind of knowledge (2D knowledge).

In the Modeling New Experiences in POMDPs section, we present the basic specifications of our computational framework and validate our method of initializing agents with prior knowledge from a previous world (e.g. initializing a 3D agent with 2D knowledge). In the Evidence for Restructuring? section, we demonstrate through computation times during training that moving from a 2D to a 3D world is a kind of change that requires an agent to effectively learn from scratch while moving to an “expanded” 2D environment enables the agent to usefully extrapolate from prior knowledge. In the What Gets Restructured? section, we show that differences in relative computation time translate to qualitative differences in the underlying agents’ policies. In the Validating the Computational Results With Human Intuitions section, we explore human intuitions regarding these moves from a 2D to 3D world. Altogether, these computational experiments provide the initial evidence for our formalization of epistemically transformative learning.

## MODELING NEW EXPERIENCES IN POMDPs

Before turning to our validation and case studies, here we present details for how we implement the basic 2D world structure in a POMDP.

### POMDP Specifications

**Figure 1.**
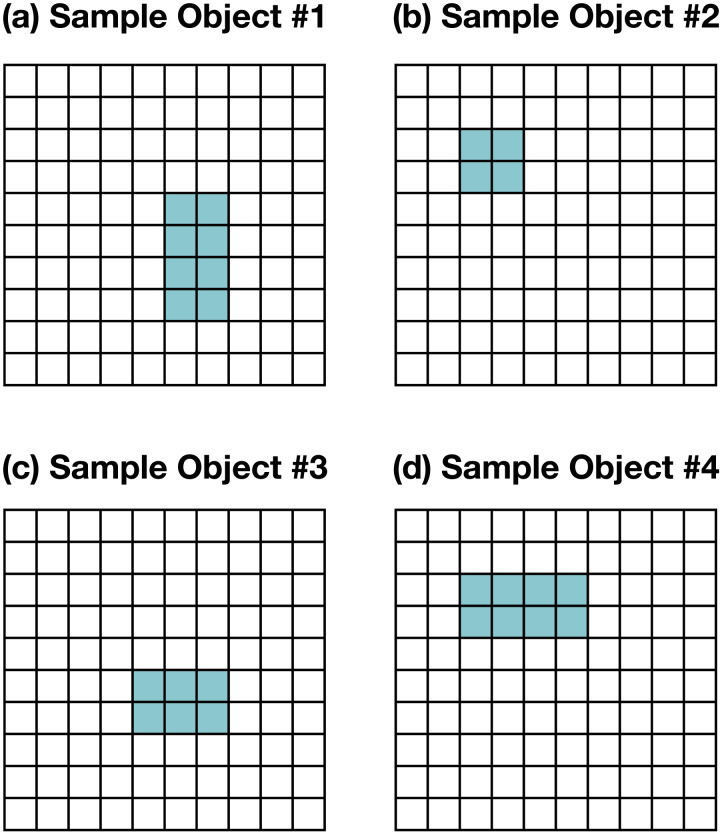
Possible objects in the two-dimensional world: (a) 2 × 4 rectangle. (b) 2 × 2 square. (c) 3 × 2 rectangle. (d) 4 × 2 rectangle.

Throughout, we consider events where an agent is navigating a 10 × 10 world that contains one of four possible objects: a 2 × 4 rectangle (Figure 1a), a 2 × 2 square (Figure 1b), a 3 × 2 rectangle (Figure 1c), or a 4 × 2 rectangle (Figure 1d). The agent’s goal is to identify which of the four objects is in the world, and it can navigate around the space to see it from different fields of view. Below we specify the details of this environment, which serves as the basis for the two case studies in the current project. The modifications of these basic settings to produce different categorical changes in the agent’s experience will be described in more detail in the respective sections (a more complete presentation is available in supplementary materials).

**Figure 2.**
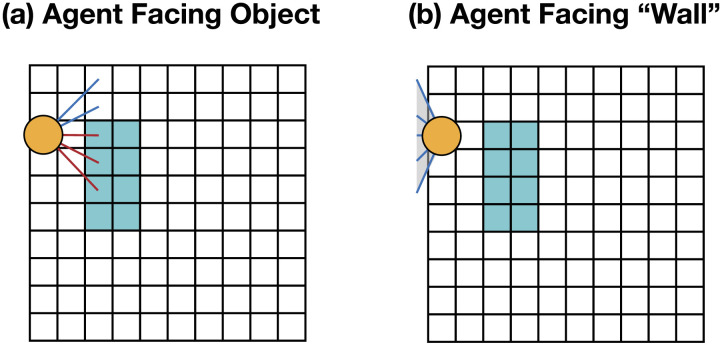
(a) The orange circle represents the agent, and the five line segments together represent the agent’s field of view. Blue segments do not intersect with any object, while red segments intersect with the object in view. (b) Initial position and orientation of the 2D-Agent.

- **State space.** In this setting, each state encodes the agent’s position in space (as an x and y coordinate) as well as the direction that they are looking at (north, south, east, or west), which of the four objects is in the scene, and its position in the 10 × 10 × 2 world. We also include a terminal state that represents that the simulation has been terminated. (And in a 2D world, the second dimension is effectively inaccessible to the agent.)
- **Action space.** The action space consists of five movement actions (move north, south, east, or west, and ‘jump’), five rotation actions (look north, south, east, or west, and ‘look down’), and four judgment actions (each a guess about which of four objects is in the world).
- **Transition function.** We use a deterministic transition function, where the agent’s actions always produce a consistent and predictable state change. Specifically, the agent’s actions are always guaranteed to be successful (e.g., the action ‘move north’ changes the world to a state where the agent has shifted one position to the north), except for when the agent attempts to walk past a physical border (e.g., trying to move north at the northern edge of the world), which produces no state change. (And in a 2D world, jumping to another dimension and looking down also produced no state changes for the two-dimensional agent.) Finally, when the agent takes a judgment action (i.e., guessing what object is in the world), the state always transitions to the terminal state. This creates a problem structure where the agent knows that it will have only one opportunity to guess the identity of the object.
- **Costs and rewards.** All movements and rotations cost −0.10. Correct guesses yielded a reward of 2000, while incorrect guesses yielded a cost of −1000. The small movement and rotation cost was introduced so that the planner favors the most efficient strategies for identifying the object. The cost and rewards of guessing were set to be disproportionately large to avoid cases where an agent might find it more rational to guess prematurely due to the cost associated with identifying the nature of the object.
- **Observation space and function.** We model the agent’s observations as a binary vector that captures the agent’s field of view. Specifically, we consider a resolution of five bits, such that the observations always consist of five binary values, each one indicating whether an object is in view or not. For instance, in Figure 2a the observation would be [0,0,1,1,1], whereas in Figure 2b it would be [0,0,0,0,0].
- **Initial beliefs.** Throughout, we assume that the agent always knows their position and orientation but does not know which of the four objects is in the scene. Formally, the probability of any state with incorrect agent information (i.e., wrong position or orientation) is initialized to 0, and a uniform distribution is used for the remaining states (i.e., the states of the world where the agent has the right location and orientation information, but where the object in the scene can be different).

### Measuring Learning

To build the policy, we used the approximate POMDP planning toolkit (APPL), which implements the SARSOP algorithm (Kurniawati et al., 2008). Throughout, we used computation time as a proxy for the amount of computation. To make all computation times comparable, each simulation was always run on an isolated dedicated core on a high-performance computing cluster. Therefore, the exact computation times will vary depending on which system the models are run on, but the relative times preserve the relative amount of computation that was necessary.

We first ran a series of cases that show how the average computation time needed for convergence works as a proxy of how much an agent’s knowledge needs to be revised. We trained a naïve agent to identify objects in a 2D world (POMDP Specifications section), then partially ablated the agent’s knowledge, and measured how long it would take to re-learn the full policy after the agent lost 25%, 50%, or 75% of its knowledge. We predicted that the stronger the ablation, the more computation time would be required to re-learn how to navigate the 2D world, therefore showing that the average computation time is a proxy for how much useful knowledge is contained in a policy.

Formally, after training the agent’s policy (i.e., training it to navigate a 2D world with the goal of identifying the object in a scene), we randomly froze a subset of the policy according to the ablation parameter (e.g., freezing 25% of the policy in the 75% knowledge-loss condition), and then scrambled all remaining values of the policy (see supplementary materials for detailed explanation), and then used this ablated policy as the starting point to re-train the POMDP, measuring the average computation time needed to fix the policy. Because the exact computation time will depend on the random subset of knowledge that is frozen, we ran each ablation study 1000 times, randomly ablating a different subset on each trial.

**Figure 3.**
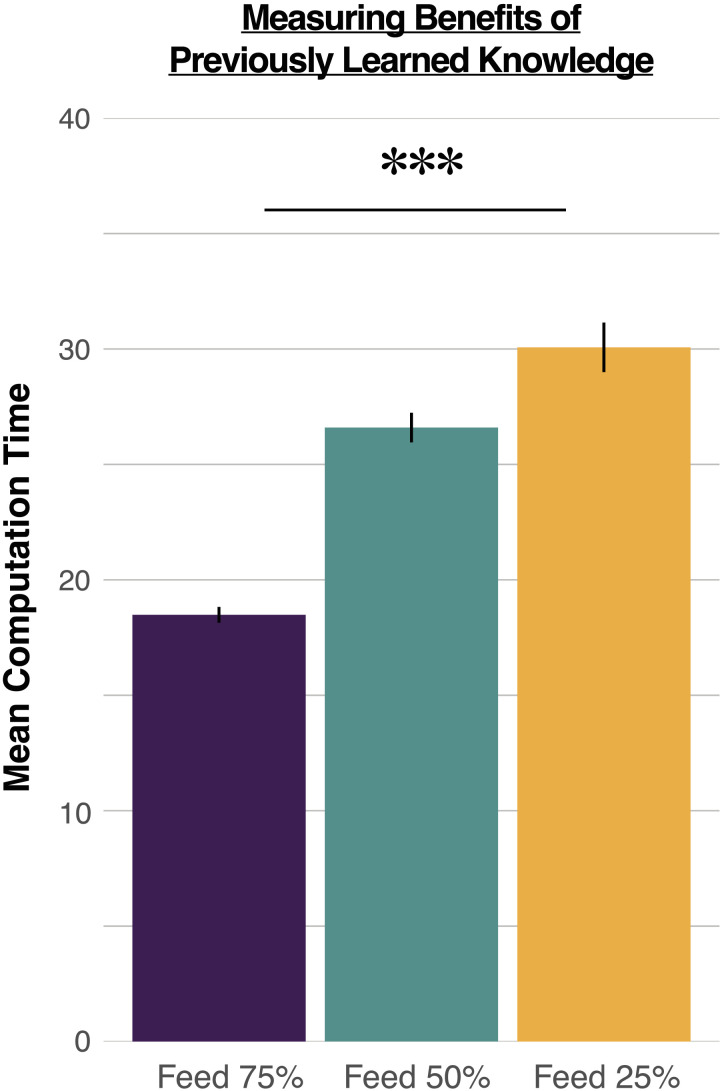
Average computation time (in seconds) for models initialized with varying percentages of the solution computed with the naïve agent. The y-axis depicts the average computation time over 1000 trials. Error bars reflect 95% confidence intervals.

Figure 3 shows the average computation time required to re-learn the policy as a function of each ablation. As predicted, average computation time increased as a function of the magnitude of the ablation. On average, the model required 30.07 s when 25% of the knowledge was retained (95% CI: 29.00–31.14), 26.60 s when 50% of the knowledge was retained (95% CI: 25.95–27.24), and 18.48 s when 75% of the knowledge was retained (95% CI: 18.14–18.83). Moreover, the amount of knowledge was a significant predictor of the average computation time needed to relearn the policy (*β* = −0.23; *p* < .001). Thus, the average computation time needed to learn a policy can capture the amount of knowledge that was useful in the previous policy.

## EVIDENCE FOR RESTRUCTURING?

We then explored whether the knowledge contained in a policy designed to identify objects in a 2D world would confer an advantage when undergoing different types of new experiences reflecting changes in the world. In one case study, an agent enters a new 3D world (“New Dimension” condition), whereas in another case study, the agent enters a new expanded 2D+ world (“Expanded World” condition). The final training structure is therefore as follows: First, we obtain the computation time needed for an agent to learn how to navigate the initial 2D world. We then test how this agent’s knowledge changes when it is either (1) transferred into a 3D world, or (2) transferred into an 2D+ world, by obtaining the computation time required for the agent to learn how to navigate the new environment. We compare this learning against an agent learning to navigate these worlds, initialized with scrambled knowledge from the original agent (to control for the amount of information stored in the agent’s policies). In particular, in the move from one world to another, new actions and observations may become exploitable by the agents in a way that was not available before the move. As such, agent policies may be dramatically different across the 3D and 2D+ worlds. Thus, it is important to interpret computation times when an agent is initiated with prior knowledge *relative* to the case when the agent is initiated with the same amount of information that is then scrambled to mimic learning from scratch. If our intuitions are correct, we expect that the computation time required for the agent to learn a policy in the 3D world should be roughly equal to the computation time required for a “scrambled” agent to learn a policy in the 3D world, thus indicating that, when moving to a 3D world, the agent’s prior knowledge (i.e., policy for navigating the 2D world) was not useful for learning to navigate the 3D world. Conversely, when moving from a 2D environment to another 2D environment with a larger grid, we expect that the “scrambled” agent should take significantly longer to learn a policy than the non-scrambled agent, indicating that the agent’s prior knowledge was useful for learning to navigate the new environment.

**Figure 4.**
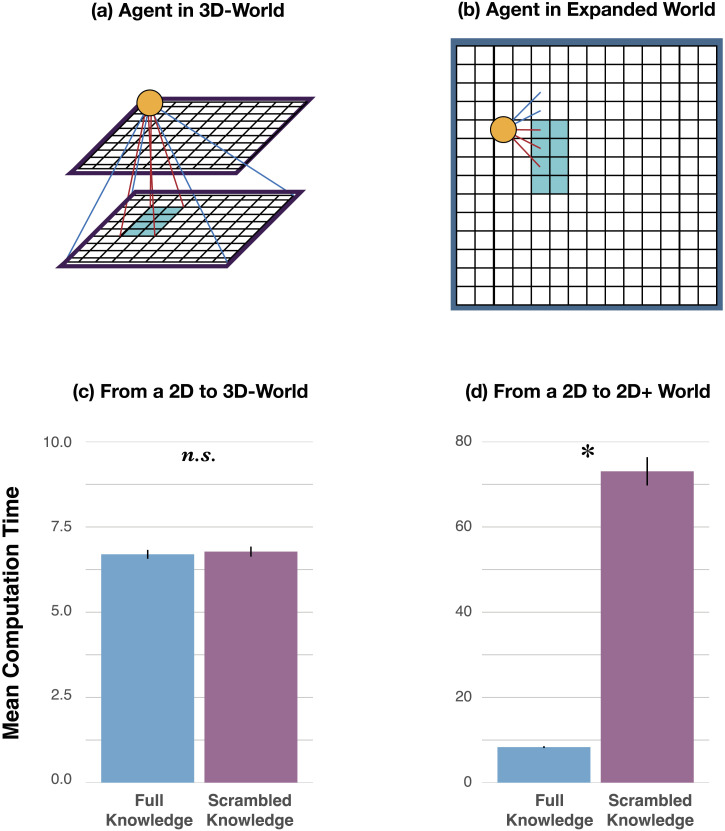
(a) An agent in a 3D world. (b) An agent in the expanded world. (c) Results in the New Dimension condition comparing agents initialized with full prior knowledge versus scrambled knowledge. (d) Results in the Expanded Dimension condition comparing agents initialized with full prior knowledge versus scrambled knowledge. The y-axis depicts the average computation time (in seconds) over 1000 trials. Error bars reflect 95% confidence intervals.

The overall method followed the logic of our validation study (Measuring Learning section). For the New Dimension condition, the agent now had access to the additional actions ‘move up’ and ‘look down’, the transition function was extended accordingly (following the same deterministic structure as in the 2D world), and the observation space was extended to include four new observations, which corresponded to the experience of seeing the object from the third dimension. These observations were only available to the agent when it jumped out of the 2D space (to the top layer of the world), and took the action ‘look down’. For the Expanded World condition, the state space was extended to cover a 12 × 12 × 2 2D world (see Figure 4b). The action space, transition function, observation space, and observation function were extended in a straightforward manner so that it followed the same principles as the simpler 10 × 10 2D world. (Thus, whereas the state space remains the same size when moving from the 2D to the 3D world [both 10 × 10 × 2], the state space increases when moving from the 2D to the 2D+ world.)

Per the final training structure we described above, we trained a naïve agent to identify objects in our basic 2D world. We then initialized the main agent in the New Dimension and Expanded World conditions with the solution of the naive agent trained in the basic 2D world. We compared the computation time of the main agents in the New Dimension and Expanded World conditions to the computation time of their scrambled knowledge agent counterparts. We predicted that the computation times of the main agent in the New Dimension condition would be no different from the computation times of its corresponding scrambled knowledge agent, indicating that the previous knowledge from the 2D world would not be useful for learning to navigate the 3D world. In contrast, the computation times of the main agent in the Expanded World condition would be shorter compared to the computation times of its corresponding scrambled knowledge agent, as the knowledge of how to navigate the 2D world should still be useful for navigating another 2D world with a larger grid. Each condition was run 1000 times to obtain a probability distribution over how much learning would be required. Altogether, this is effectively a 2 × 2 design, where we look at computation times as a function of change (New Dimension vs. Expanded World) and knowledge (Full vs. Scrambled).

Figure 4c–d shows the average computation time required to re-learn the policy as a function of change (New Dimension vs. Expanded World) and type of knowledge (Full vs. Scrambled). In the New Dimension condition (Figure 4c), we found no signature of extrapolation from prior knowledge, such that average computation time when the main agent was initialized with prior knowledge was not reliably different from the average computation time when the main agent was initialized with scrambled knowledge (6.70 s [95% CI: 6.57–6.83] vs. 6.78 s [95% CI: 6.63–6.92], *t*(1998) = 0.79, *p* = .429). That is, an agent with full competence acting in a 2D world takes the same amount of time to learn how to act in a 3D world as an agent that had no prior competence. This was in contrast to results from the Expanded World condition (Figure 4d), in which average computation time significantly decreased when the main agent was initialized with full prior knowledge, compared to the average computation time when the main agent was initialized with scrambled knowledge (8.35 s [95% CI: 8.14–8.56] vs. 73.05 s [95% CI: 69.74–76.37], *t*(1998) = 48.29, *p* < .001).

Such a comparison might seem striking, given it only takes around 6–7 s for the agent to learn in the 3D world, across both the full knowledge and scrambled knowledge cases, compared to almost 80 seconds to learn a policy from scratch in the 2D+ world (i.e., the scrambled knowledge case). But the discrepancy can be explained by going back to the main (epistemic) goal of the agent in this world, which is to learn what objects are in the world it is in. In light of this goal, an agent in the 3D world needs only to jump up to the new dimension and look down from a bird’s eye view. This is in contrast to an agent in a 2D (and 2D+) world that needs to move around the (even expanded) world to learn what objects are in the world. In this case, the agent with competence acting in a 2D world learned how to act in the expanded world over eight times faster than an agent that lacked that competence.

These results altogether show that, while knowledge from the 2D world was useful for the agent in the 2D+ world, it did not provide any benefit when the agent moved to the 3D world. An agent discovering a distinctively new kind of experience is effectively “learning from scratch.” But this does not mean that the agent in the 3D world is necessarily worse off, since moving to the 3D world unlocked new abilities (such as jumping up and looking down) for the 3D agent that were initially not useful for the agent when it was just in the 2D world. In other words, these new abilities facilitated the agent’s goal of learning what objects are in its world. We can relate this result back to the example of how seeing red for the first time can be epistemically transformative—in that it may unlock new abilities (e.g., being able to separate the red socks from the green socks for the first time while doing laundry). These results show that our framework can capture the idea that a change from a 2D to a 3D environment is epistemically transformative, whereas the expansion of a world (with a similar structure) is not.

## WHAT GETS RESTRUCTURED?

Thus far, we have suggested that a change in the agent’s knowledge of the world (e.g., gaining access to a new kind of perceptual experience via 3D information) is epistemically transformative to the degree that it requires an agent to effectively learn their policy from scratch. We showed this by demonstrating that, after gaining access to 3D information, the agent’s prior knowledge (a policy for navigating the 2D world) was not useful for learning to navigate the 3D world: the agent that started with a 2D policy took just as long to learn a 3D policy as the agent that started with a completely scrambled policy.

Note, however, that our learning-time measure does not reveal the *content* of what the agent learns after gaining 3D experience, only that the original 2D policy is not useful for learning a 3D policy (but *was* useful for learning a policy in the expanded 2D+ world). While this satisfies our main goal to identify an empirical signature that indicates when and to what degree a change in experience is epistemically transformative, it is worth asking what exactly the 3D agent is learning, and what is distinctive about this knowledge as compared to the 2D+ agent. That is, what is it about the 3D policy that renders the agent’s prior knowledge (original 2D policy) so useless? Here we propose a way of comparing two different policies for navigating the same environment. We use this additional measure to explore how the observed differences in learning time reflect actual underlying changes in the agent’s policy.

To this end, it is necessary to introduce a few more details about how POMDPs represent knowledge internally. In the standard POMDP framework, an agent’s policy—i.e., its knowledge about how to act—is encoded by a function that assigns a value to each action under any possible knowledge state. Intuitively, if the agent knows the true state of the world *s*, then the value of taking action *a* in state s reflects how much “closer” to the goal the agent will be after taking action *a*: the closer a gets you to a rewarding state from state *s*, the higher its value in state *s*. In a POMDP, however, the agent does not know the true state of the world, and represents their uncertainty as a probability distribution P(S) over possible world states, which gets updated by the agent’s new observation after each action. Thus, for a POMDP agent, the value of taking action *a* in *belief state* P(S) reflects an *expectation* about how close action *a* will get the agent to the goal state. Importantly, “closeness” in a POMDP can reflect both spatial proximity (e.g., getting physically closer to a goal object) *and* epistemic proximity (e.g., becoming more certain about where a goal object is located). In our 2D task, for example, the agent’s goal is to correctly guess which object is in the grid, so the value of taking action *a* in belief state P(S) is closely tied to the expected reduction in uncertainty that would result from the ensuing observation.

Given a value function that assigns a value to each action x belief-state pair, the optimal policy is to deterministically choose the action with the highest value in each belief state. While this is useful in some domains like robotics (where the concern is getting an agent to behave optimally in an environment), these fully optimized, deterministic agents are less useful for developing descriptive models of actual human behavior, which is frequently noisy and suboptimal. To relax this assumption, we can create a probabilistic policy that allows an agent to make different choices from the same belief state. This is commonly achieved by transforming the value function over actions into a probability distribution using SoftMax (see Jara-Ettinger et al., in press; for details).

A SoftMax function converts a vector of real numbers into a vector of probabilities in such a way that preserves the ordinality of the original numbers (i.e., if v = (a, b) and SoftMax(v) = (p_a_, p_b_), then p_a_ > p_b_ if and only if a > b). Thus, under a SoftMax policy, actions with higher expected values are more likely to be selected than actions with lower expected values. Importantly, the softMax function includes a *temperature* parameter (call it *T*) which controls how deterministic the policy is. For very small values of *T*, the policy is nearly deterministic, meaning that the agent will almost always choose the action with the highest expected value. When T is very large, the policy is nearly uniformly random, so that each action is equally likely to be selected. While the exact value of *T* is not important for the analysis that follows, the crucial point is that we can represent the agent’s policy as a set of probability distributions over actions, one for each possible belief state, and that the probability associated with each action is directly correlated to the expected value of that action (given the belief state). This allows us to compare two different policies for the same POMDP world by comparing the probability distributions over actions in corresponding belief states.

For our purposes, we must characterize three different comparisons. First, we must characterize how the agent’s policy changes after we modify the environment from 2D to 3D. Second, we must characterize how the agent’s policy changes after we modify the environment from 2D to 2D+ (by expanding the grid). Finally, we must characterize how the change in policy from 2D to 3D differs from the change in policy from 2D to 2D+. To make the comparisons between policies, we use Kullback-Liebler (KL) divergence, a standard measure of divergence between probability distributions. Intuitively, if P and Q are two different probability distributions over actions, then KL(P, Q) reflects the “surprisal” of an agent who believes that distribution P represents the best way to act observing the behavior of an agent who believes that distribution Q is the best way to act.

**Figure 5.**
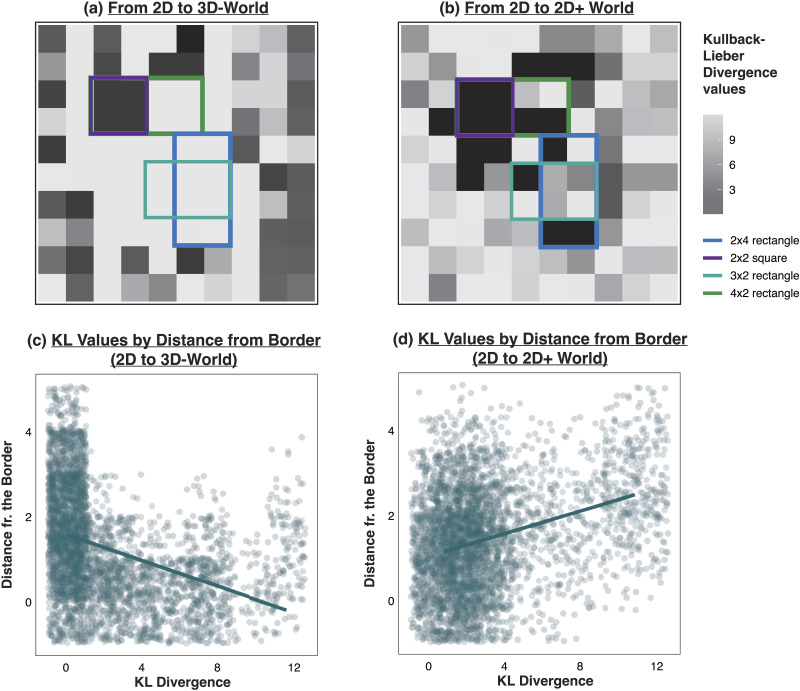
(a) KL divergence values for each state comparing action distributions of the 2D-agent versus the 3D-agent (darker blue reflects lower KL divergence, while lighter blue reflects higher KL divergence). Rectangular outlines reflect the different possible objects in the world. Note that action distributions are defined for each *belief* state, so the value in each cell reflects the average KL divergence overall belief states corresponding to that cell. (b) KL divergence values for each state comparing action distributions of the 2D-agent versus the 2D+ agent. (c) KL divergence values for the 2D-agent vs. the 3D-agent, plotted as a function of distance from the border. KL divergence decreases as a function of distance from the border. (d) KL divergence values for the 2D-agent vs. the 2D+ agent, plotted as a function of distance from the border. KL divergence decreases as a function of distance from the border.

With this established, our analysis works as follows. We first quantify the difference between the original 2D policy and the newly learned 3D policy by computing, for each belief state, the KL divergence between the 2D action distribution and the 3D action distribution, and then repeat this using the 2D policy and 2D+ policy. Figure 5a–b depicts the average KL divergence value for each cell in the environment (recall that KL divergences are computed for each *belief state*, so to visualize these values in a 2D grid, we average KL divergences across all belief states within the same grid location). A quick inspection of this figure already reveals clear differences in *where* the changes in the agent’s action distributions are occurring in different locations around the grid. In particular, for the 2D vs. the 3D agent (Figure 5a), the action distributions differ the most for belief states around the border of the grid (shown as lighter blue squares on the edges)—which is in contrast to the 2D vs. 2D+ agent case (Figure 5b), where the action distributions differ the most for the central states. This difference was further verified by plotting the KL divergence values in the 3D-agent and 2D+ agent cases against the distance of the state from the border. This shows an inverse relationship, where KL divergence decreases with distance from the border in the 3D-agent case (Figure 5c; Pearson’s *r* = −.41, *p* < .001), but increases with distance from the border in the 2D+ agent case (Figure 5d; Pearson’s *r* = .45, *p* < .001).

This analysis suggests that *where* the agent is on the grid matters for understanding the change in the agent’s policy. To recall our introductory example, where our colorblind agent is suddenly able to see the color red, it seems plausible that this change in their experience would have the greatest impact on their behavior in states of the world where the newfound ability to see red is relevant: if they are reading a black-and-white newspaper, it seems unlikely that being able to see red would significantly alter how they approach this task. On the other hand, if our agent is navigating a fruit market and trying to determine which pieces of fruit are ripe, the ability to see red would enable a host of new strategies for achieving this goal, and would likely have a much greater impact on their behavior. Thus, in addition to characterizing how the agent’s policy changes within each state, our analysis should also reflect how important each state is to the agent’s task: changes to the policy in more task-relevant states should be more indicative of epistemic transformation than changes to the policy in task-irrelevant states.

**Figure 6.**
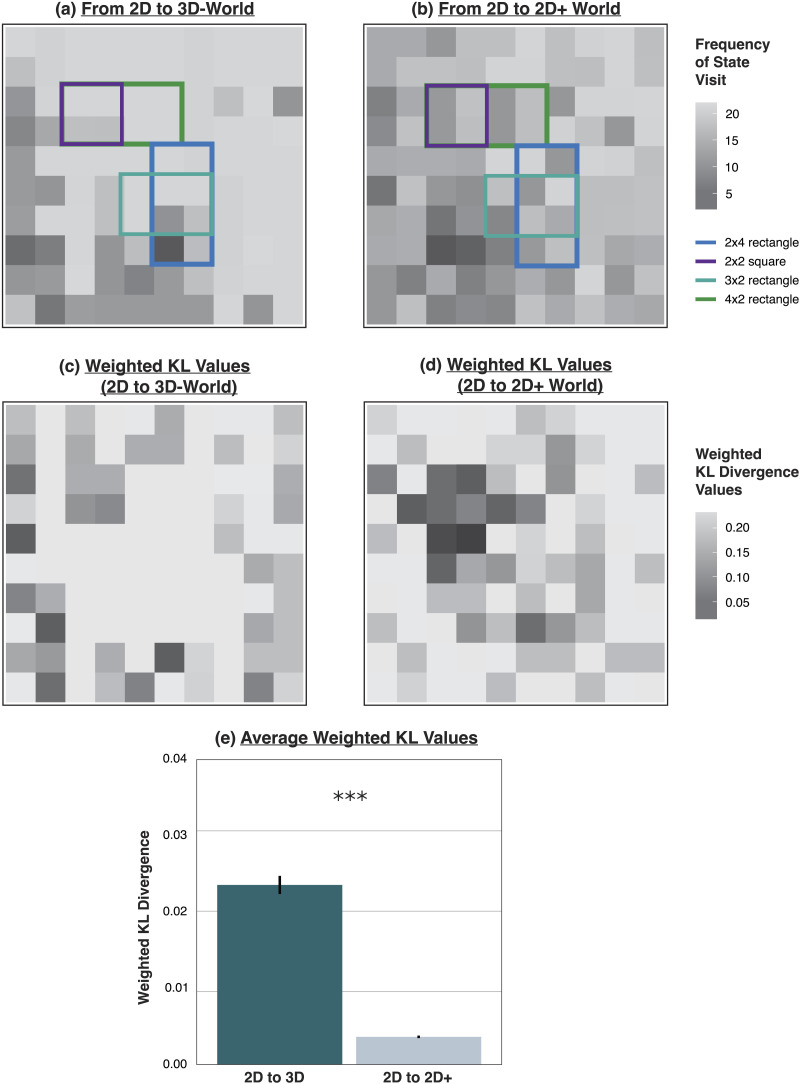
(a) Frequencies with which the particular state was visited in the 3D-world. (b) Frequencies with which the particular state was visited in the 2D+ world. (c) KL divergence values weighted by state visit frequency for the 2D-agent vs. the 3D-agent. (d) KL divergence values weighted by state visit frequency for the 2D-agent vs. the 2D+ agent. (e) Mean weighted KL values in the 2D vs. 3D-agent contrast versus the 2D vs. 2D+ agent contrast. Mean KL divergence was significantly greater when moving to a 3D-world, compared to a 2D+ world.

To obtain a proxy measure for how “task-relevant” a particular state is, we estimated how frequently the agent visited each state under each policy by selecting a random starting point for the agent 500 times and simulating the agent’s policy forward until completion. This yielded a tally of how many times each agent visited each state, depicted in Figure 6a–b. Since the POMDP agent navigates by probabilistically selecting higher-value states, the frequency with which the agent visits a given state can be interpreted as a proxy for how “important” that state is to its goals. In our object identification task, the agent has access to a wider, unobstructed viewpoint of the object from cells closer to the edge of the grid than from cells closer to the center of the grid, reflected in Figure 6a–b by the higher visit frequencies along the left and bottom borders as compared to the center. Thus, cells closer to these edges are higher-value with respect to this task. Figure 6c–d shows the same KL divergence values from Figure 5a–b, with each value weighted by the frequency with which that state was visited.

To obtain a final measure of how much the policy changed when moving from a 2D to 3D world, we took the KL divergence between action distributions for each state, and averaged these values across states, weighting each value by the frequency with which the agent visited that state. This gives us an aggregate measure of how much the agent’s policy changed when moving from 2D to 3D, with more “important” states being more heavily weighted than less “important” states. We then repeated this procedure for the agent that moved from a 2D to a 2D+ world, to enable a direct comparison between the amount of policy change experienced when moving to a 3D world and the amount of policy change experienced when moving to an expanded 2D+ world. This analysis reveals that there is a significant difference between the average KL divergence when weighted by state frequency visit in the 3D-agent case than in the 2D+ case (Figure 6e; *M* = .024 vs. *M* = .004, *t*(7996) = 32.48, *p* < .001), suggesting a greater degree of policy change in the 2D-to-3D case as compared to the 2D-to-2D+ case.

Altogether, our initial analysis in the Evidence for Restructuring section revealed that going from a 2D to a 3D world involves an epistemically transformative change in the sense that the 2D agent’s prior knowledge conferred no advantage when re-learning a policy for the 3D world. The frequency-weighted KL divergence measure in this section further suggests that this re-learning is occurring for the higher-value border states that the agent visits more frequently (and are therefore more important to solving the task). Thus, this analysis reveals something further about where the agent’s knowledge is being restructured: in the 2D to 2D+ case, changes to the agent’s policy are concentrated in the center of the grid, but the higher-value (and more frequently visited) states are those on the border. When moving from 2D to 2D+, the agent is primarily changing the way in which they visit central cells to border cells. In the 2D to 3D case, however, changes to the policy are concentrated at the edge of the grid. The 2D to 3D agent is primarily restructuring how they behave when they are in the high-value border states, rather than expanding their strategy beyond the border states.

## VALIDATING THE COMPUTATIONAL RESULTS WITH HUMAN INTUITIONS

In a separate online experiment, we explored how these computational results might align with human intuitions (further information is available in the supplementary materials). Subjects were first introduced to the concept of ‘Flatland’ and were asked to “imagine that they are a circle” in this 2D world. To ensure that subjects understood the context, they were asked to rate their understanding of the world and the instructions from 1–4, with 1 being “did not understand at all”, and 4 being “I got that completely!”. They were then presented with the two possible worlds (3D world vs. the expanded world). Using a slider in which they could move a disc between “World 1” (the 3D world) and “World 2” (the expanded world), they were asked to compare these two possible worlds along three dimensions:

1)  *Imagination*. Subjects were asked: “Now, imagine you’ve lived in the 2D world all your life, and have never visited any of the possible worlds. What would be harder to imagine: what it’s like to live in World 1, or what it’s like to live in World 2?”
2)  *Action*. Subjects were asked: “Now imagine that suddenly you discovered that you could actually try living in these possible worlds for a day. Which experience would have a larger change on how you would act, such as where you would go or what you would do?”
3)  *Description*. Subjects were asked: “After coming back to the two-dimensional world, you now want to tell other people about these possible worlds that you experienced. Which world/experience would be more difficult to describe to your friends who have lived in the 2D world all their lives?”

**Figure 7.**
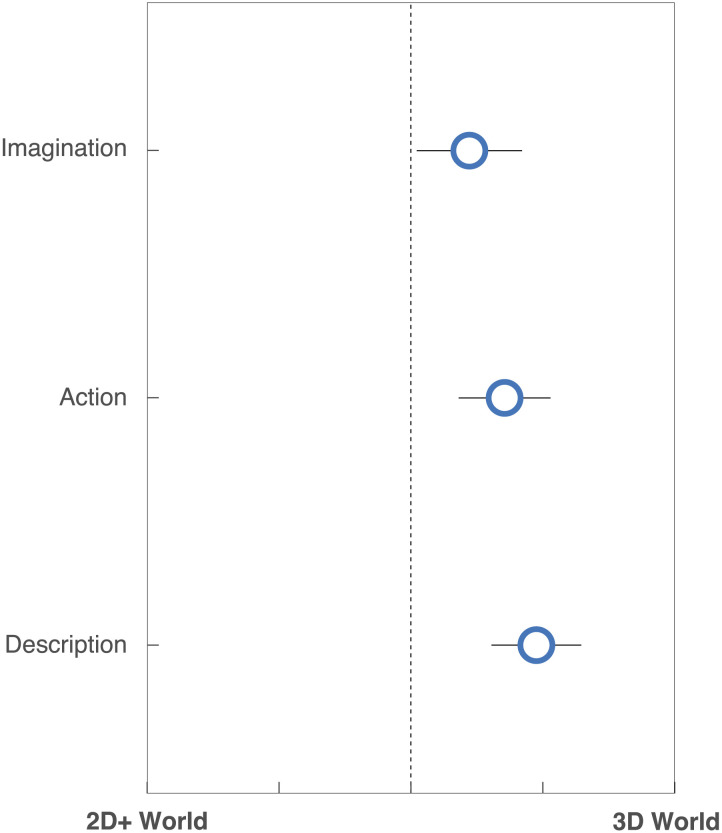
Mean subject ratings for each of the question types. The y-axis depicts the question type, and the x-axis depicts the slider position (as being rated closer to the 2D+ world vs. the 3D world). Error bars reflect 95% confidence intervals.

Subjects reported an average rating of 3.72/4 (*SD* = 0.57) in the comprehension questions, suggesting that they grasped the context of ‘Flatland’. Mean subject ratings are depicted in Figure 7. For all three questions, subjects moved the slider reliably more towards the 3D-world (Imagination: *t*(49) = 2.18, *p* = .034; Action: *t*(49) = 4.00, *p* < .001; Description: *t*(49) = 5.48, *p* < .001), and there was a main effect of question type (*F*(1, 49) = 6.74, *p* = .012). Thus, people’s responses support the intuition that the 3D world is more challenging to describe, imagine, and adapt to—suggesting an epistemically transformative experience.

## GENERAL DISCUSSION

The POMDP framework has been useful for explaining many aspects of human thought and behavior (e.g., Griffiths et al., 2019; Lieder & Griffiths, 2020; Ongchoco et al., 2024). Here we use it to formalize a distinctive shift in an agent’s knowledge based on the kind of new experience they have. This is important because, for some new experiences, such as considering what an avocado-raspberry milkshake tastes like, knowledge of what this combination tastes like can merely be inferred from pre-existing knowledge and experience (of what avocados taste like, of what raspberries taste like, and of what milkshakes taste like). In contrast, for other new experiences, such as what it would be like for a colorblind individual to see red, knowledge of what it’s like requires experience of a new kind. We thus asked whether we could formalize this distinction as a signature of an “epistemically transformative” change.

Our formalization involved the setting up of a “Flatland”-inspired grid world, in which an agent initialized in a 2D-world experienced a move either to an expanded 2D+ world or to a 3D-world. We predicted the former would reflect the first type of merely “expanded” experience (akin to tasting an avocado-raspberry milkshake when one already knows what avocados, raspberries, and ordinary milkshakes taste like) while the latter would reflect the second, new type of experience (akin to a colorblind individual learning what it’s like to see red). When going from a 2D to a 3D world, policy computation times in the new environment seemed to reflect the agent “learning from scratch”, suggesting that existing knowledge did not support learning in the new environment, presumably because the new knowledge could not simply be inferred from prior knowledge. This is the signature of an epistemically transformative experience that we propose. In contrast, when going from a 2D to an expanded 2D world, policy computation times were faster when initialized with previous knowledge, suggesting that existing knowledge did facilitate learning in the new environment (even when the state space was larger in the expanded 2D world).

In what ways might prior knowledge not be helpful for the acquisition of new knowledge? One way to understand the differences in learning times is to consider where the relearning was happening for the agent in the 3D world. For this, we sought to compare the policies of agents pre- and post-experience (e.g., the policy of a 2D agent versus a 3D agent). Previous work on comparing POMDP policies has largely focused on comparing the performance of the policies, rather than the knowledge encoded in those policies. In the absence of an established method, we directly compared the action distributions in corresponding belief states. With this method, we first found that the difference between action distributions of an agent who has undergone an epistemically transformative change (going from 2D to 3D) was reliably greater than the difference between the action-value distributions of an agent who did not. We then found that places of learning (where there was the greatest difference in action distributions) corresponded to positions in the grid world that the agent visits to pursue its goal of identifying objects in the environment. This suggests that this measure of difference depends on the structure of the agent’s task and environment: if the agent exists in an environment where perceiving the color red does not enable any new strategies or behaviors, then this new form of experience would not drastically alter the agent’s policy.

### Future Directions

A key contribution (and the main challenge) of this work is to blend computational work with philosophical theories. The current work presents a very specific approach towards understanding epistemically transformative experiences. By formalizing it this way, it becomes clearer exactly what is predicted or explained by the framework, but also where the model can be improved. As a result, we hope our work will inspire new questions and new research formalizing these experiences. We can think of at least three immediate future directions that one can explore: (1) the kinds of epistemic changes agents might experience, (2) the ways agents might encounter these changes; and (3) the space of possible worlds the agent might consider when updating their policies.

For example, the case studies we explored here generally involved “upgrades” in experience, in which a new kind of knowledge was gained—whether an agent went from a 2D to a 3D world, or a colorblind individual gained sight after an operation. But epistemic changes could also include cases where knowledge and abilities are lost—such as when an agent goes from a 3D to a 2D world, or an individual loses sight. Future work could run similar simulations on the *reversal* of the cases we explore here. And more generally, as we only explored two case studies of possible transformative vs. non-transformative experiences in the context of an artificially created world, a more thorough follow-up study could apply our framework to a graded range of POMDPs (e.g., adding incrementally more dimensions, more colors, more cells to the grid, etc.), and compare our measures of “transformativeness” against human judgments for the same range of worlds. It remains an open question how our computational framework might capture people’s intuitions across a broader range of experiences.

Moreover, in the current work, the movement of the agent from one world to another was directly manipulated, and so was the initializing of their policies with policies learned from their previous environments. In this sense, the agent was effectively dropped into a new world, forced to take on any relearning necessary to navigate the new environment. But in everyday experiences, transformative experiences are not always sudden. Moreover, in some instances, we can prepare more for impending transformative experiences. One might ask: when are policies relearned and replaced *in anticipation* of the experience? To explore this question, a computational framework would need to integrate a “meta-model” that decides when it might be optimal to relearn and replace the policy.

Finally, epistemically transformative learning relates to counterfactuals, as it concerns possibilities that an agent has not experienced and cannot imagine well enough to know what they are like. The current framework can be extended to represent this idea. For example, one could potentially define notions of “imaginability” and “unimaginability” in terms of possible worlds, and incorporate this into the agent’s broader decision process (in line with the previous point about “meta-models”) about when and how to update versus replace their existing policy.

### Conclusions

Ultimately, these differences in the learning patterns of an agent undergoing a change in a grid world are useful for thinking about our own epistemic transformations. We mention three broad topics here. First, epistemically transformative experiences, as discussed in philosophy, lead to major changes in value functions. Our framework may help us better understand and distinguish between different types of value change: those in which a core value function is added or replaced, and those in which a core value function is merely developed. The types of transformative experiences that are of most interest often involve ones where some core value functions are added or replaced. (For example, as Paul (2014) argues, when a mother gives birth to a child, or a colorblind individual experiences red for the first time.) Second, the differences in learning patterns observed here (in particular, how the discovery of something new may come with the need to re-learn one’s policy) may explain, to some degree, variations in how people respond to transformative experiences or learn new concepts (e.g., Amsel, 1992; King et al., 2017). Moreover, learning and relearning of new concepts may also occur not just on the individual level (as in moments of insight or discovery of new knowledge; Hélie & Sun, 2010; Kounios & Beeman, 2014; Smith, 1995), but also on the social or scientific level (as in a political or scientific revolution; Kuhn, 1970; Paul, 2014). Finally, recent work explores questions that our framework could also bear on, for example, when, in response to epistemic transformation, one restructures their value function after a religious conversion (e.g., Markovic, 2022; Paul, 2021), or gains a new kind of understanding after becoming a parent (e.g., Crone, 2021; Molouki et al., 2020). As such, our computational approach provides a new experimental framework for exploring the different ways in which transformative experiences can change us.

## ACKNOWLEDGMENTS

We thank Elizabeth Bonawitz, Rachit Dubey, and two other anonymous reviewers for helpful comments.

## FUNDING INFORMATION

JJE was supported by NSF award IIS-2106690.

## AUTHOR CONTRIBUTIONS

J. D. K. Ongchoco and I. M. Davis worked on the computational framework with input from J. Jara-Ettinger. J. D. K. Ongchoco, I. M. Davis, J. Jara-Ettinger, and L. A. Paul wrote the manuscript.

## DATA AVAILABILITY STATEMENT

Code and data for the simulations and behavioral experiment are available in the OSF repository: https://osf.io/3q8a4/?view_only=b226ed07d7894945b5e7467b842a701b.

## Note

^1^ The classic Jackson (1986) version of this thought experiment concerns Mary, a “super-knower” who knows all the scientific truths that could be known, living in a highly stylized context where we have reached the end of all scientific enquiry, and where, in particular, she knows every physical truth about the brain that could be known. The debate is thus focused on whether Mary, as a super-knower, can infer the phenomenal truths from all the physical truths she knows, without leaving her black and white room. This is not the context we are exploring: we are interested in real life contexts, so our Mary is a rather “ordinary Mary” who knows as much about the world as, say, a contemporary scientist knows. We also note that we are merely using the phrase “what it’s like” as a synonym for “phenomenal character”: we are not invoking or endorsing the existence of “qualia.” (We thank a referee for pressing us to make these clarifications.)

## Supplementary Material


