# Supplementary material for: When New Experience Leads to New Knowledge: A Computational Framework for Formalizing Epistemically Transformative Experiences
Source: Open Mind (Camb). 2024 Nov 1;8:1291–311. doi: 10.1162/opmi_a_00168 (PMC11563650; doi:10.1162/opmi_a_00168)
Supplement: Supplementary file 1 [file opmi-08-1291-s001.pdf]

Supplementary Materials

Basic POMDP Implementation Details

To implement the experiment design formally, we constructed a world in which there were one of four possible objects: a 2x4 rectangle, a 2x2 square, a 4x2 rectangle, or a 3x2 rectangle. Each state encodes the agent's position in space as well as the direction that they are looking at, which of the four objects is in the scene, and its position in the 10x10 grid world. Finally, we include a terminal state that represents that the simulation has been terminated. Thus, our task state space consisted of 4000 states (100 possible positions x 2 dimensions x 5 possible orientations x 4 possible objects), and a terminal state.

In a 2D world, the agent could perform a movement action (move north, south, east, or west), or a rotation action (look north, south, east, or west). In a 3D world, the agent could also 'jump' to the other dimension, and 'look down' — but these actions were disabled for the 2D agent (such that performing these actions incurred a cost of –1000, and did not bring the 2D agent into a different state in the state space). The agent could also perform judgment actions (each consisting of a guess about which of the four objects is in the grid-world). If an agent correctly guessed the object, then it received an observation of "correct", and if not, then it received an observation of "wrong". Thus, there were a total of 14 actions in our action space.

At any given point as the agent is moving or rotating in its world, it can see only within a particular range of cells, depending on its location and orientation. We formalized what the agent can see at any given moment as a string of 5 digits (e.g., 00000), with each digit representing a line segment drawn from the agent's position to one of five cells in the direction it is facing. If any of these five line segments intersected with an object, the corresponding digit of the 5-digit string was then changed to 1. So if two line segments intersected with an object, the 5-digit string might look something like 00011. If an agent tried to move past the borders of the grid (e.g., if it moves left even though it is already in the leftmost column of the space), or if it

faced these borders (e.g., if it faces west even though it is in the leftmost column of the space), it received a blank observation (00000). The agent had perfect vision — which means the agent always received the right observation 100% of the time.

Each movement and rotation action incurred a subjective cost of –0.10. Correct guesses yielded a reward of 2000, while incorrect guesses yielded a reward of –1000. The model used a discount parameter of 0.95.

Agent in the 2D world. To verify that our set-up yields to different agent policies, depending on what object exists in the world, we then computed the policies of the agent when there was a 2x4 rectangle, a 2x2 square, a 4x2 rectangle, or a 3x2 rectangle. The agent was initialized at the upper right corner of the grid world. When there is a 2x4 rectangle in the world, the agent first 'looks east', then 'moves south' four times, then 'moves east' two times, and then provides an answer. When there is a 2x2 rectangle in the world, the agent first 'looks east', then 'moves east' twice, and then provides an answer. When there is a 4x2 rectangle in the world, the agent first 'looks east', then 'moves east' twice, and then provides an answer. When there is a 3x2 rectangle in the world, the agent first 'looks south', then 'moves south' four times, then 'moves east' two times, and then provides an answer.

Agent in the 3D world. The set-up is exactly as noted in the 2D world, except now, the action of 'jumping' brought the agent to the 3rd dimension (i.e., a different state in the state space), and looking down allowed the agent to see a wider 'bird's-eye' view of the dimension below — formalized as a string of 60 digits.

Agent in the expanded grid world. The set-up is exactly as noted in the 2D world, except now, the state space comprises of a 12x12 grid world, rather than a 10x10 grid world. The task state space consisted of 5760 states (144 possible positions x 2 dimensions x 5 possible orientations x 4 possible objects), and a terminal state.

Feeding Previously Computed Solutions to the POMDP

Any policy computed from a POMDP is captured as a list of alpha-vectors, each associated with an action. These alpha-vectors encode information about how useful each action is under different beliefs. The algorithm that we used (i.e., the SARSOP algorithm; Kurniawarti et al., 2008) modifies the policy over multiple iterations by adding or pruning alpha-vectors from the list, until the changes are small enough that it reaches a precision level of 0.001. Thus, the number of iterations needed to compute the policy (and the corresponding amount of time that this takes) gives us a measure of how difficult it was to compute a final list of alpha-vectors.

Within the SARSOP algorithm, we introduced several modifications. First, we set-up a switch in which the SARSOP algorithm could use either the default lower bound (which determines the initial list of alpha-vectors it starts with), or a custom lower bound — which would be defined by the pre-computed solution that we feed to the solver. Second, we set-up two custom parameters when feeding the solver with a pre-computed solution: (1) a "vector-cap" parameter, and (2) a "shuffle" parameter. When the "vector-cap" parameter is called, a random N proportion of alpha-vectors is preserved (e.g., 25%, or 100%) and fed straight to the solver. When the "shuffle" parameter is called, the remaining alpha-vectors from (1) that were *not* preserved are then shuffled in two ways. First, values within each alpha-vector are shuffled. Second, values in each alpha-vector are shuffled across the alpha-vectors (essentially re-assigning values to a different alpha-vector — so that the association between alpha-vectors and actions is effectively scrambled). When the lengths of the alpha-vectors differ (as a result of differing sizes of state spaces), values are randomly selected from the full list of values from the alpha-vectors shuffled above, and used to then fill in the remaining values of the alpha-vectors.

Validating the Case Study with Human Intuitions

Participants. 50 observers from the United States were recruited using Prolific online platform. This sample size was determined before data collection began, was pre-registered, and fixed to be identical across the experiments reported here. All experimental methods and procedures were approved by the Yale Institutional Review Board, and all subjects confirmed

that they had read and understood a consent form outlining their risks, benefits, compensation, and confidentiality, and that they agreed to participate in the experiment.

Stimuli and Procedure. Subjects were first introduced to the world of "Flatland", and asked to imagine that they are a circle in this 2D-world. Subjects were given examples of what different shapes in this world would look like. They were then asked to rate their understanding of the world and the instructions from 1-4, with 1 being "did not understand at all", and 4 being "I got that completely!". Afterwards, they were presented with descriptions of two possible worlds: "In World 1, there is a third dimension that you can 'jump' onto, such that you can view shapes on a plane from above. And in World 2 (as on the right), there is a larger space of the world that you can move into, such that you can view shapes from nearer or further distances." Using a slider in which they could move a disc between "World 1" and "World 2" (with positions of the worlds randomly determined across questions and subjects — such that World 1 could appear on the left while World 2 could appear on the right side of the slider, or vice versa), they were then asked to compare these two possible worlds along three dimensions:

1) *Imagination.* Subjects were asked: "Now, imagine you've lived in the 2D world all your life, and have never visited any of the possible worlds. What would be harder to imagine: what it's like to live in World 1, or what it's like to live in World 2?"
2) *Action.* Subjects were asked: "Now imagine that suddenly you discovered that you could actually try living in these possible worlds for a day. Which experience would have a larger change on how you would act, such as where you would go or what you would do?"
3) *Description.* Subjects were asked: "After coming back to the two-dimensional world, you now want to tell other people about these possible worlds that you experienced. Which world/experience would be more difficult to describe to your friends who have lived in the 2D world all their lives?"

Results. Subjects reported an average rating of 3.72/4 (*SD*=0.57) in the comprehension questions. For all three questions, subjects moved the slider reliably more towards the 3D-world (Imagination: $t(49)=2.18$, $p=.034$; Action: $t(49)=4.00$, $p<.001$; Description: $t(49)=5.48$, $p<.001$), and there was a main effect of question type ($F(1, 49)=6.74$, $p=.012$).
